# *Arhgap22* Disruption Leads to RAC1 Hyperactivity Affecting Hippocampal Glutamatergic Synapses and Cognition in Mice

**DOI:** 10.1007/s12035-021-02502-x

**Published:** 2021-08-28

**Authors:** Anna Longatti, Luisa Ponzoni, Edoardo Moretto, Giorgia Giansante, Norma Lattuada, Maria Nicol Colombo, Maura Francolini, Mariaelvina Sala, Luca Murru, Maria Passafaro

**Affiliations:** 1 Institute of Neuroscience, CNR, Milan, 20129 Italy; 2 Department of Pharmacological and Biomolecular Sciences, Università Degli Studi Di Milano, 20133 Milan, Italy; 3 NeuroMI Milan Center for Neuroscience, Università Milano-Bicocca, 20126 Milan, Italy; 4 Department of Medical Biotechnology and Translational Medicine, Università Degli Studi Di Milano, 20129 Milan, Italy

**Keywords:** ARHGAP22, Hippocampus, Dendritic spines, Synaptic plasticity, Learning and memory

## Abstract

Rho GTPases are a class of G-proteins involved in several aspects of cellular biology, including the regulation of actin cytoskeleton. The most studied members of this family are RHOA and RAC1 that act in concert to regulate actin dynamics. Recently, Rho GTPases gained much attention as synaptic regulators in the mammalian central nervous system (CNS). In this context, ARHGAP22 protein has been previously shown to specifically inhibit RAC1 activity thus standing as critical cytoskeleton regulator in cancer cell models; however, whether this function is maintained in neurons in the CNS is unknown. Here, we generated a knockout animal model for *arhgap22* and provided evidence of its role in the hippocampus. Specifically, we found that ARHGAP22 absence leads to RAC1 hyperactivity and to an increase in dendritic spine density with defects in synaptic structure, molecular composition, and plasticity. Furthermore, *arhgap22* silencing causes impairment in cognition and a reduction in anxiety-like behavior in mice. We also found that inhibiting RAC1 restored synaptic plasticity in ARHGAP22 KO mice. All together, these results shed light on the specific role of ARHGAP22 in hippocampal excitatory synapse formation and function as well as in learning and memory behaviors.

## Introduction

The Rho family of small GTPases (Rho GTPases) is a class of signaling G-proteins known to orchestrate several cellular processes, such as cell migration, vesicle trafficking, and differentiation [1]. Moreover, it has been shown that Rho GTPases control actin cytoskeleton dynamics [2] and therefore regulates CNS synapses [3].

The most extensively studied members of this family in the nervous system are RHOA, RAC1, and CDC42 [4]. While the former is involved in the formation of actin-myosin contractile fibers and focal adhesion, RAC1 and CDC42 play a key role in regulating actin polymerization through the activation of several downstream proteins, such as actin-related protein 2 (ARP2), serine/threonine-protein kinase 1 (PAK1), and Wiskott-Aldrich syndrome protein family member verprolin-homologous protein (WAVE). In addition, these proteins act in concert to exert their functions through reciprocal regulation [5]. In particular, PAK1 is able to directly bind active RAC1 triggering a signal cascade that involves different effectors modulating actin polymerization [6, 7]. Moreover, WAVE is recruited by RAC1-GTP at the plasma membrane and in turn it activate ARP2, a part of the ARP2/3 complex which works as a nucleator platform for the formation of new actin filaments [8, 9].

Rho GTPases act as molecular switches cycling between active and inactive states, when bound to GTP or GDP, respectively [10, 11]. Two classes of proteins are known to regulate Rho GTPases activity: (1) guanine nucleotide exchange factors (GEFs) that promote GDP exchange with GTP and are considered positive modulators and (2) GTPase activating proteins (GAPs) that enhance the intrinsic activity of Rho GTPases thereby inactivating them [12]. Misregulated RHOA, RAC1/RAC3, and CDC42 activity have been associated with cognitive disorders [13–17].

The *arhgap22* gene, located on the chromosome 10 in humans and 14 in mice, encodes for ARHGAP22 protein (also called RhoGAP2 and RhoGAP22), a member of the ARHGAP family. This protein is structurally composed of N-terminal pleckstrin-homology (PH) domain, followed by RhoGAP and C-terminal coiled-coil (CC) domains. ARHGAP22 is ubiquitously expressed in mammals, with a prevalence in highly vascularized tissues [18], and has been demonstrated to specifically inhibit RAC1 through its GAP activity [18, 19], thus standing as a critical cytoskeleton regulator. Previous reports have linked ARHGAP22 activity to cell movement and morphology as well as to actin dynamics in cancer, through its RAC1 regulating activity [19, 20]. In addition, ARHGAP22 has also been proposed as cellular transcription regulator [18]. Previously, we found that ARHGAP22 is localized at post-synaptic site of the excitatory synapses and interacts with interleukin-1 receptor accessory protein-like 1 (IL1RAPL1) protein in order to induce dendritic spines formation [21].

In this study, we showed that ARHGAP22 is expressed in the CNS peaking during synaptogenesis. The absence of *arhgap22* in mouse brain causes alterations in excitatory synaptic structure, function, and in mice behavior. We also observed that *arhgap22* loss of function induces RAC1 hyperactivation and the treatment with NSC23766, an inhibitor of RAC1 activity, rescues some of the defects.

These findings establish the important role of ARHGAP22 protein in excitatory synapse formation with key repercussions on cognitive functions.

## Material and Methods

### Generation of arhgap22 KO Mice

*Arhgap22* KO mice were generated using gene-trapping technique. Mice (strain C57BL/6) were generated from an embryonic stem (ES) cell line (IST13119C7 clone, Texas Institute for Genomic Medicine, TIGM). The retroviral (Omni- Bank Vector 76) cassette contained a splice acceptor sequence (SA) followed by a 5′ selectable marker –geo (a functional fusion between the beta galactosidase and neomycin resistance genes) for identification of successful gene trap events, followed by a poly-adenylation signal (pA). Insertion of the retroviral vector into the *arhgap22* gene (intron 3) led to the splicing of the endogenous first 3 exons and the cassette to produce a truncated transcript of *arhgap22* gene. 3′ RACE was used to verify the insertion of the cassette into the correct genomic location. The ES cell clone, containing the retroviral cassette in the Arhgap22 gene, was microinjected into C57BL/6 host blastocysts to generate chimeras using standard procedures. Chimeric males were bred to C57BL/6 wild type females for germ line transmission of the knock-out Arhgap22 allele.

### Real-Time PCR

mRNA was extracted from murine tissues using Nucleozol Reagent (Macherey Nagel) following manufacturer’s instructions. A total of 1.5 µg of extracted mRNA was used to synthetize cDNA using SuperScript™ VILO™ cDNA Synthesis Kit (Thermo Fisher). Following, *arhgap22* specific sequence was amplified from 60 ng of cDNA in the presence of SYBR Green PCR Master Mix (Applied Biosystems) using an Applied Biosystems 7000 Real-Time thermocycler. Parallel PCR reaction was performed with α-actin-specific primers as housekeeping control gene.

The sequences of primers (Sigma Aldrich) were the following: *arhgap22* Fw97 (TTCGGCCACAGATAGAGGAT), *arhgap22* Rev97 (GTCATCAGATGCTGAACCAGAG), α-actin Fw (AGATGACCCAGATCATGTTTGAGA), α-actin Rev (CCTCGTAGATGGGCACAGTGT).

Each sample was run in triplicate, and the results were analyzed by ABI PRISM 7000 software using the ΔΔCT method to allow the normalization of each sample to the internal standard and comparison with the calibrator.

### Rac1-GTP Pulldown Assay

pGex-PakCRIB construct (kind gift from G. Pietrini) was used to produce GST-CRIB fusion protein. GST fusion protein was prepared in BL21 *Escherichia coli* and purified using standard procedures [22]. To evaluate the levels of active RAC1, we performed the Rac1-GTP pulldown assay. Briefly, hippocampal and cortical samples from adult mice were collected and homogenized in RAC Buffer (10% glycerol, 100 mM NaCl, 10 mM MgCl2, 50 mM Tris HCl pH 7.4, 10% triton, protease inhibitor). Lysates were then centrifuged at 12000* g* for 10 min at 4 °C. Supernatants were incubated with 40 μl of S-transferase PAK glutathione-coupled Sepharose 4B beads (GE Healthcare) for 30 min at 4 °C. After 3 washes with RAC buffer, beads were resuspended in sample buffer (4% SDS, 10% 2-mercaptoethanol, 20% glycerol, 0.004% bromophenol blue, 0.125 M Tris–HCl). Bound protein as well as inputs were loaded on polyacrylamide gels and analyzed by Western blotting.

### Crude Synaptosomes Preparation

Cortices and hippocampi were dissected from *arhgap22* WT and KO adult animals as previously reported [23] and homogenized with a glass-teflon homogenizer in cold HEPES/sucrose buffer (4 mM HEPES [pH 7.4], 0.32 M sucrose, protease inhibitor). After centrifugation at 1000* g* at 4 °C for 10 min, Supernatant (S1) were centrifuged at 10000* g* at 4 °C for 15 min. Resulting supernatant contained mainly cytosolic components while the pellet was composed of crude synaptosomes (P2). After resuspension in 10 volumes of HEPES/sucrose buffer, pellet was centrifuged at 10000* g* for 15 min at 4 °C to purify the preparation. P2 fractions were resuspended in modified RIPA buffer (50 mM TRIS–HCl, 200 mM NaCl, 1 mM EDTA, 1% NP40, 1% Triton X-100, pH 7.4, protease inhibitor cocktail) and then quantified with bicinchoninic acid assay (BCA) (Euroclone) prior to SDS-PAGE and Western blotting.

### F/G Actin Ratio

To study F/G ratio, we followed a previously described protocol [24]. Briefly, hippocampal and cortical samples from adult mice [23] were resuspended in actin lysis buffer (10 mM K2HPO4, 100 mM NaF, 50 mM KCl, 2 mM MgCl2, 1 mM EGTA, 0.2 mM DTT, 0.5% Triton, 1 mM sucrose [pH 7]) and centrifuged at 15000* g* for 30 min at 4 °C. Supernatants containing G-actin were collected while the pellet was resuspended in 1 volume of actin lysis buffer/1 volume of Buffer II (1.5 mM guanidine hydrochloride, 1 mM sodium acetate, 1 mM CaCl2, 1 mM ATP, 20 mM Tris HCl [pH 7.5]). After 1-h incubation on ice, samples were centrifuged at 15000* g* for 30 min at 4 °C. Supernatant containing F-actin was collected. G-actin and F-actin samples were then resuspended in sample buffer prior to Western blotting analyses.

### SDS-PAGE and Western Blot

Samples were loaded on polyacrylamide gels at different concentrations (7.5–15%) and then transferred onto nitrocellulose membrane (0.22 μm, GE Healthcare). Membranes were incubated, at room temperature for 2–3 h or overnight at 4 °C in Blocking buffer (non-fat dry milk 5% and TBS Tween-20), with the primary antibodies against the following protein categories: (1) RAC1 and downstream effectors (α-RAC1 (Abcam, 1:500), α-WAVE (SantaCruz Biotechnology,1:500); α-ACTIN (Sigma Aldrich,1:4,000); α-PAK1 and pPAK1 (Cell Signaling, 1:1000), α-ARP2 (SantaCruz Biotechnology,1:500)); (2) α-TUBULIN (Sigma Aldrich,1:40,000) and α-GAPDH (Santa Cruz Biotechnology, 1:2000) as loading controls; (3) subunits of glutamatergic receptors (α-NR1 (Gift from C. Gotti, 1:500), α-NR2A (Sigma Aldrich, 1:1500), α-NR2B (Chemicon, 1:1000); α-GLUA1 (Chemicon, 1:1000); α-GLUA2/3 (Gift from C. Gotti, 1:2500); α-GLUK2 (Sigma Aldrich 1:1000)); (4) post-synaptic proteins (α-CASK1 (Neuromab 1:2500); α-PSD-95 (Neuromab, 1:2500), α-PICK1 (Neuromab; 1:1000), α-GRIP1 (BD transduction laboratories, 1:2000)); and (5) pre-synaptic markers (α-VGAT (SySy, 1:1000), α-synaptophysin (SySy, 1:5000)). After washing, the blots were incubated at room temperature for 1 h with α-rabbit or α-mouse IR Dyes- (LI-COR) conjugated antibodies (1:7500) in Blocking buffer accordingly to the host species of primary antibody. Immunoreactive bands were visualized by Odyssey Infrared Imager (LI-COR).

### Golgi and DiI Staining

We performed Golgi staining using FD Rapid GolgiStainTM Kit (FD Neurotechnologies Inc.) according to manufacturer’s instructions. Adult *arhgap22* WT and KO male mice (at least 3 *per* genotype) were deeply anesthetized with isoflurane and transcardially perfused with saline solution (0.9%). Brains were then immersed in impregnation solution for 14 days in the dark at room temperature and subsequently in solution C for 3 days at room temperature. The brains were cut in 100-μm coronal sections with a vibratome (Leica VT1000S). Slices were mounted on gelatin-coated glasses and maintained at room temperature in the dark, allowing them to dry naturally. Sections were then washed in MilliQ water and placed in solution D for 10 min. Subsequently, slices were washed and dehydrated with increasing concentrations of ethanol (50, 70, 95, and 100%) for 4 min each and, as last step, with xylene (3 times, 4 min each). At the end of the procedure, slices were mounted with a coverslip using Permount mounting medium (Fisher Scientific). Acquisition of the stained neurons from the hippocampus was performed using a Leica DMI6000 spinning disk microscope (Leica). Stacks were collected every 0.5 μm using × 63 objective. Analysis of the dendritic spine density was calculated on 100 μm dendrites/neuron using NeuroStudio software.

For DiI analyses, *arhgap22* WT and KO mice (at least 3 *per* genotype) were deeply anesthetized with isoflurane and transcardially perfused with 1.5% paraformaldehyde solution (PFA). Brains were incubated in 1.5% PFA ON at 4 °C and then cut in 3-mm coronal sections with a vibratome (Leica VT1000S). Dil 1,1′-dioctadecyl-3,3,3′,3′-tetramethylindocarbocyanine perchlorate (“DiI”; DiIC18(3)) crystals were placed on top of each slice immersed in PBS overnight. A total of 100-μm coronal sections were cut with a vibratome (Leica VT1000S) and mounted with a coverslip using Fluoromount mounting medium (Sigma Aldrich). Acquisition of the stained neurons from the hippocampus was performed using a Zeiss 510 confocal microscope (Carl Zeiss). Stacks were collected every 0.5 μm using × 63 objective. Analysis of the dendritic spine density and spine morphology were performed on 100 μm dendrites/neuron using NeuroStudio software using parameters to define mushroom, stubby, and thin spines as in [25].

### Electron Microscopy

Adult *arhgap22* WT and KO male mice (3 *per* genotype) were deeply anesthetized with isoflurane before transcardial perfusion with EM buffer (2.5% glutaraldehyde and 2% paraformaldehyde in 0.15 M sodium cacodylate buffer, pH 7.4). The brains were further fixed in EM buffer for 24 h at 4 °C. A total of 100 μm slices were obtained with a vibratome (Leica VT1000S) and hippocampi manually dissected. Samples were then post-fixed with 2% osmium tetroxide, rinsed, en bloc stained with 1% uranyl acetate, dehydrated, and embedded in epoxy resin (Electron Microscopy Science, Hatfield, PA, USA). Thin Sects. (70–90 nm) were obtained with EM UC6 ultramicrotome (Leica Microsystems, Austria), stained with a saturated solution of uranyl acetate in ethanol 20%, and observed under a Philips CM10 transmission electron microscope (TEM) (FEI, Eindhoven, Netherlands). Analyzed excitatory synapses (apical dendrite layer of the hippocampal CA1 region) were identified on the base of three conditions: (1) the presence in their postsynaptic terminal of the electron-dense post-synaptic density (PSD); (2) the presence of at least 3 synaptic vesicles in the pre-synaptic compartment; (3) the presence of a defined synaptic cleft. Images were acquired at a final magnification of × 25–34,000 using a Morada CCD camera (Olympus, Munster, Germany) and analyzed with ImageJ software (NIH Image).

### Electrophysiology

Adult *arhgap22* WT and KO male mice (at least 3 per genotype) were used for electrophysiological recordings. Coronal hippocampal slices (thickness, 300/400 μm) were prepared as previously described [26] using a vibratome VT1000S (Leica). After the cutting procedure, brain slices were incubated in aCSF (125 mM NaCl, 2.5 mM KCl, 1.25 mM NaH2PO4, 1 mM MgCl2, 2 mM CaCl2, 25 mM glucose, and 26 mM NaHCO3; pH 7.3, equilibrated with 95% O2 and 5% CO2) for 40 min at 35 °C for miniature excitatory and inhibitory post-synaptic currents (mEPSCs/mIPSCs) or at room temperature for field excitatory post-synaptic potentials (fEPSPs) recordings. mEPSCs and mIPSCs were recorded from CA1 pyramidal neurons in voltage-clamp mode in aCSF supplemented with lidocaine (500 µM) to block voltage-gated sodium channels, and using an internal solution containing (in mM) 130 CsGluconate, 8 CsCl, 2 NaCl, 10 HEPES, 4 EGTA, 4 MgATP, and 0.3 Tris-GTP (pH 7.4). The excitation/inhibition (E/I) balance was calculated through the ratio between mEPSCs and mIPSCs amplitudes recorded from the same neuron [27].

fEPSPs were evoked through Schaffer collateral (SC) stimulation (0.05 Hz of frequency) and recorded from the *stratum radiatum* of the hippocampal CA1 using aCSF-filled capillaries.

Input–output (I–O) relationship was obtained measuring the slope of the fEPSPs evoked in response to increased stimulation intensity (0–1.0 mA). Stimulus strength was adjusted to give 50% maximal response and long-term potentiation (LTP) was induced by high-frequency stimulation (HFS) (100 stimuli at 100 Hz).

For paired pulse ratio (PPR) experiments, pairs of stimuli were delivered at 50-ms intervals every 20 s (0.05 Hz) and the ratio was calculated by dividing the amplitude of the second response by the first one [28]. Currents and fEPSPs were filtered at 2 kHz and digitized at 20 kHz using Clampex 10.1 software through the patch-clamp amplifier. All the analyses were performed offline with Clampfit 10.1 software.

### Microelectrode Array

Adult mice were anesthetized with isoflurane prior to decapitation. Coronal hippocampal slices (400 μm thick) were cut using a vibratome (Leica VT1000S) in an ice-cold high-sucrose protective solution containing (in mM) 87 NaCl, 25 NaHCO_3_, 2.5 KCl, 0.5 CaCl_2_, 7 MgCl_2_, 25 glucose, 75 sucrose, and saturated with 95% O_2_ and 5% CO_2_. Before recordings, slices were left to recover for at least 1 h at room temperature in aCSF. APS-MEA has been previously described [29, 30]. Recordings were performed at room temperature and slices were continuously perfused at a rate of 4–5 ml/min with aCSF. After a slice stabilization period (at least 30 min), 4-amynopyridine (4-AP; 100 μm, Sigma-Aldrich) was applied for at least 30 min before activity recordings (5 min per session). All the analyses were conducted off-line using BrainWave software (3Brain Gmbh, Switzerland). Hippocampal activation clusters were defined manually according to mouse brain atlas. A *band*-*pass filter* (from 100 to 250 Hz) was applied to the raw data to extract extracellular local field potentials (LFPs). Spikes and LFPs were detected and analyzed through hard threshold (HT) algorithm (spikes, threshold − 100 μV; refractory period, 1 ms; LFPs, high threshold 100 μV; low threshold, − 100 μV; with maximum LFP event duration < 1 s).

### Behavioral Tests

All the behavioral tests were conducted on adult *arhgap22* WT and KO male mice (6 to 10 per genotype), and depending on the type of test, we replicated the experiments 2/3 times. Animals were kept in 12-h light/dark cycle with food and water ad libitum.

#### General Health Assessment

Animals were evaluated once a week to check their general health status by measuring their body weight and food intake.

#### Spontaneous Motor Activity

Mice locomotion integrity was evaluated by recording animals in automated activity cage (43 × 43 × 32 cm) (Ugo Basile, Varese, Italy). Cumulative horizontal and vertical beam breaks were counted for 180 min [31].

#### Balance Beam

Balance beam test was used to assess mice balance and coordination. Mice were trained and tested to cross beams of different widths (6 and 12 mm). Latency to traverse each beam was recorded [32].

#### Pole Test

In the vertical pole task, the mouse was placed on a vertical wire-mesh pole with its head facing upwards. Mice were habituated to the task in 2 trials per day for 2 days. On test day (third day), mice were subjected to 5 trials: the total time taken to turn the body and to descend was recorded according to the literature [33]. A cut-off of 60 s was given. Data were shown as mean of 5 trials evaluated during the test day.

#### Wire Hanging

Muscle strength was evaluated by positioning the mouse on the top of a wire cage lid. After shaking the lid to give the mouse the possibility to grip to the wire, the operator turned upside down the lid. Latency to fall off the wire was recorded [34].

#### Novel Object Recognition

Novel object recognition test was divided into two distinct phases: the familiarization phase and the object recognition phase. After 10 min of habituation in a cage, two different objects were placed at the center of the arena and mice were free to explore the objects for 20 min. After the familiarization phase, animals were returned to the home cage. The experiment was repeated at different time points (5 min, 120 min, and 24 h later) but every time, one of the familiar object was replaced by a novel one. Object recognition was achieved when mice stayed within 0.5 cm from the novel object with the nose toward the object [35].

#### Spatial Object Recognition

Spatial object recognition test was divided into two distinct phases: the familiarization phase and the spatial object recognition phase. After 10 min of habituation in an open-field arena, two different objects were placed at different corners of the arena. Mice were able to explore the objects for 20 min. After the familiarization phase, animals were returned to the home cage. After 5 and 120 min, animals were put again in the arena but every time, one of the two object was relocated [36]. The discrimination index was calculated as described in [37].

#### T-maze

T-maze test was performed to evaluate hippocampal-dependent spatial working memory in rodents. Before the experiment, mice experienced food deprivation until they had reached 85–90% of their free-feeding body weight. Then, animals were placed in the start arm of a maze with a T shape. After leaving the start arm, mice could choose between entering either the left or the right arm to explore the maze. In the acquisition phase, one arm (left or right) was used as the reinforcing area as it contained food. The percentage of animals and number of days to reach the criterion (80% of correct choices for 3 days) was calculated. Each mouse that met the criterion for acquisition was then tested using a reversal phase in which the reinforcer arm was changed [31].

#### Morris Water Maze

Water maze paradigm was performed as described in [38]. The maze consisted in a tank (diameter 1.5 m) containing water and a hidden platform placed about 1 cm below the water surface. The pool was divided into four quadrants: NW, NE, SW, and SE as reference. Several posters and furniture that provided visual cues were mounted near the maze. During the acquisition phase, mice began the experiment from different points of the pool, while the platform position was maintained (4 trials/day for 4 days). Twenty-four hours after the last trial, the platform was removed from the pool and a probe test was performed. Escape latency during acquisition phase and the time spent in the target zone of the maze during probe phase were recorded.

#### Elevated Plus Maze

Elevated plus maze was used to evaluate anxiety-related behavior. It is based on an apparatus with a cross shape: two opposite open arms and two opposite enclosed arms separated by a central platform. The apparatus was 50 cm far from the floor and it was placed in a quiet room. After 20 min adaptation to the new environment, mice were placed individually in the center of the apparatus facing an open arm. The number of open- and total-arm entries and the time spent in open arms were recorded for 5 min [39].

#### Marble Burying

Marble burying test was used to evaluate anxiety-like behavior in mice. Briefly, animals were placed in a standard cage filled with 5-cm depth of bedding and 12 marbles regularly interspersed. The number of marble buried with bedding and the latency to bury were measured [40].

### Mice Pharmacological Treatment

Adult *arhgap22* WT and KO male mice (at least 3 per group), housed in groups of maximum 4 per home cage and maintained on a 12-h light/dark cycle with food and water ad libitum, received intraperitoneal (IP) injection once a day for 5 days (at 8:00–9:00 AM). The *arhgap22* WT and KO mice weight were daily checked before the treatment. *Arhgap22* WT mice were treated with saline as a control, while KO mice were treated with saline or the RAC1 inhibitor NSC23766 (Tocris Bioscience; 2,5 mg/kg) [41]. *Arhgap22* KO mice were randomly assigned to the saline or the NSC23766 group. Mice received the last IP injection 2 h before the experiments.

### Statistical Analyses

All the experiments were performed using at least 3 animals *per* genotype. Data were expressed as mean ± standard error of the mean (SEM). Statistical analyses were performed using Prism 6 software (GraphPad, San Diego, CA). Statistical significance was determined either by Student *t* test or ANOVA followed by Bonferroni post hoc tests. A *p*-value lower than 0.05 was considered statistically significant (*p < 0.05, **p < 0.01, ***p < 0.001).

### Animal Use Guidelines

All the experiments followed the guidelines established by the Italian Council on Animal Care and were approved by the Italian Government decree No. 747/2015-PR. All efforts were made to minimize the number of subjects used and their suffering.

## Results

### Arhgap22 Transcript Is Present in Mouse Brain

**Fig. 1:**
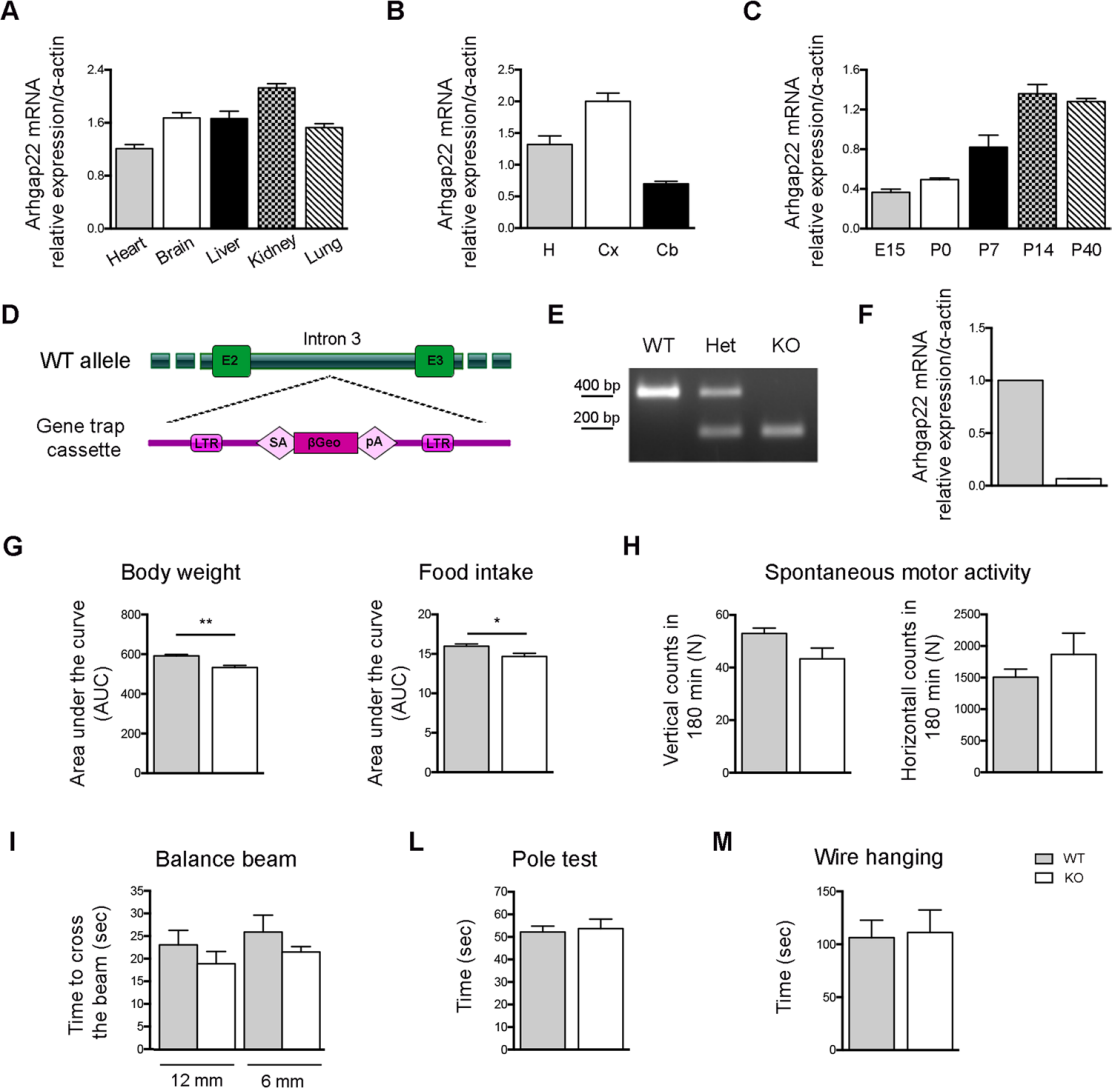
ARHGAP22 expression in mouse brain and generation of *arhgap22* KO mouse. **A** *Arhgap22* mRNA relative expression in different murine organ. ARHGAP22 is particularly expressed in the kidney, liver, and brain. Values are normalized on α-actin. Error bars indicate ± s.e.m.; **B** *Arhgap22* mRNA expression in different brain areas of adult mice. *Arhgap22* transcript is expressed in the cortex and hippocampus while it is present at lower levels in the cerebellum. Values are normalized on α-actin. Error bars indicate ± s.e.m.; **C** *Arhgap22* mRNA expression at different time-points: embryonal day 15 (E15), post-natal day 0 (P0), 7 (P7), 14 (P14), 40 (P40). The highest degree of expression is present at P14 and P40. Values are normalized on α-actin. Error bars indicate ± s.e.m. **D** Schematic representation of the vector used to randomly insert the gene-trap cassette into *arhgap22* wild type locus (intron 3). The gene-trap cassette includes the following elements: 5′ and 3′ flanking long terminal repeats (LTR), splicing acceptor (SA), βGeo marker (βGal and Neo fusion), and a polyadenylation site. **E** Characteristic genotyping PCR bands of the resulting phenotypes. Arhgap22Fw/Arhgap22Rev genotyping primer pairs hybridize on intron 4 at either side of the insertion point resulting in amplification only from the wild-type allele, whereas theArhgap22/V76R pairs result in amplification from the gene-trap cassette. **F** *Arhgap22* mRNA expression in WT and KO mice brain. KO mice show the almost complete absence of *arhgap22* transcript. Values are normalized on α-actin. Error bars indicate ± s.e.m. *Arhgap22* WT and KO mice have been tested to assess their general health state. **G** KO mice present decrease in body weight and food intake. *Arhgap22* WT e KO mice have been tested to evaluate also general motor functions. No differences were detected in spontaneous motor activity (H), balance beam (I), pole test (L), and wire hanging (M)

Since little is known about ARHGAP22 expression in the murine organism, we decided to evaluate *arhgap22* transcript levels by RT-PCR. First, we analyzed *arhgap22* transcript in different murine organs, showing that *arhgap22* is expressed in all the analyzed samples including the brain (Fig. 1A). To assess whether the transcript was present at similar levels in different brain areas, we performed RT-PCR on cortical, hippocampal, and cerebellar lysates showing that ARHGAP22 is expressed in all the regions checked with predominance in cortex and hippocampus (Fig. 1B).

We therefore used cortical and hippocampal samples to study temporal distribution of *arhgap22* transcript and we found an increase in the expression during development, reaching the highest expression levels at P14 which are then maintained stable in adulthood (Fig. 1C).

Importantly, the increase in Arhgap22 transcript levels observed at P14 coincides with the temporal window in which synaptogenesis is most prominent [42], thus suggesting that ARHGAP22 might play a role in synapse formation.

### Arhgap22 KO Mice Present Hyper-Activation of the RAC1 Pathway in Cortex and Hippocampus

To test this hypothesis and gain additional insights on Arhgap22 function in the CNS, we used a KO mouse for *arhgap22* generated by using gene-trapping strategy (Fig. 1D). To confirm the disruption of the functional *arhgap22* gene in KO mice, mutant and normal alleles were amplified by PCR. The specific *arhgap22* normal allele (400 bp band) was detected in WT and heterozygous animals, while mutated product (200 bp band) was amplified in heterozygous and KO mice (Fig. 1E). We also evaluated *arhgap22* transcript levels in cortical and hippocampal lysates from WT and KO mice by RT-PCR. Quantification demonstrated that *arhgap22* mRNA was almost completely absent in KO mice (Fig. 1F).

Since this animal model was never characterized before, we evaluated *arhgap22* KO mice general health. Although adult KO mice presented a reduction in food intake (area under the curve (AUC)l WT 15.980 ± 0.284 vs KO 14.660 ± 0.402; *n*_mice_ = 4–5, Student *t* test * *p* < 0.05) that led to a slight reduction in body weight (area under the curve (AUC); WT 591.600 ± 7.419 vs KO 552.700 ± 10.730; *n*_mice_ = 4–5, Student *t* test * *p* < 0.05) (Fig. 1G), they did not show alterations in spontaneous motor activity (Fig. 1H), balance beam (Fig. 1I), or in pole and wire hanging test (Fig. 1L, M). Taken together, these results suggest that Arhgap22 KO mice have a good general health, comparable to WT animals.

**Fig. 2:**
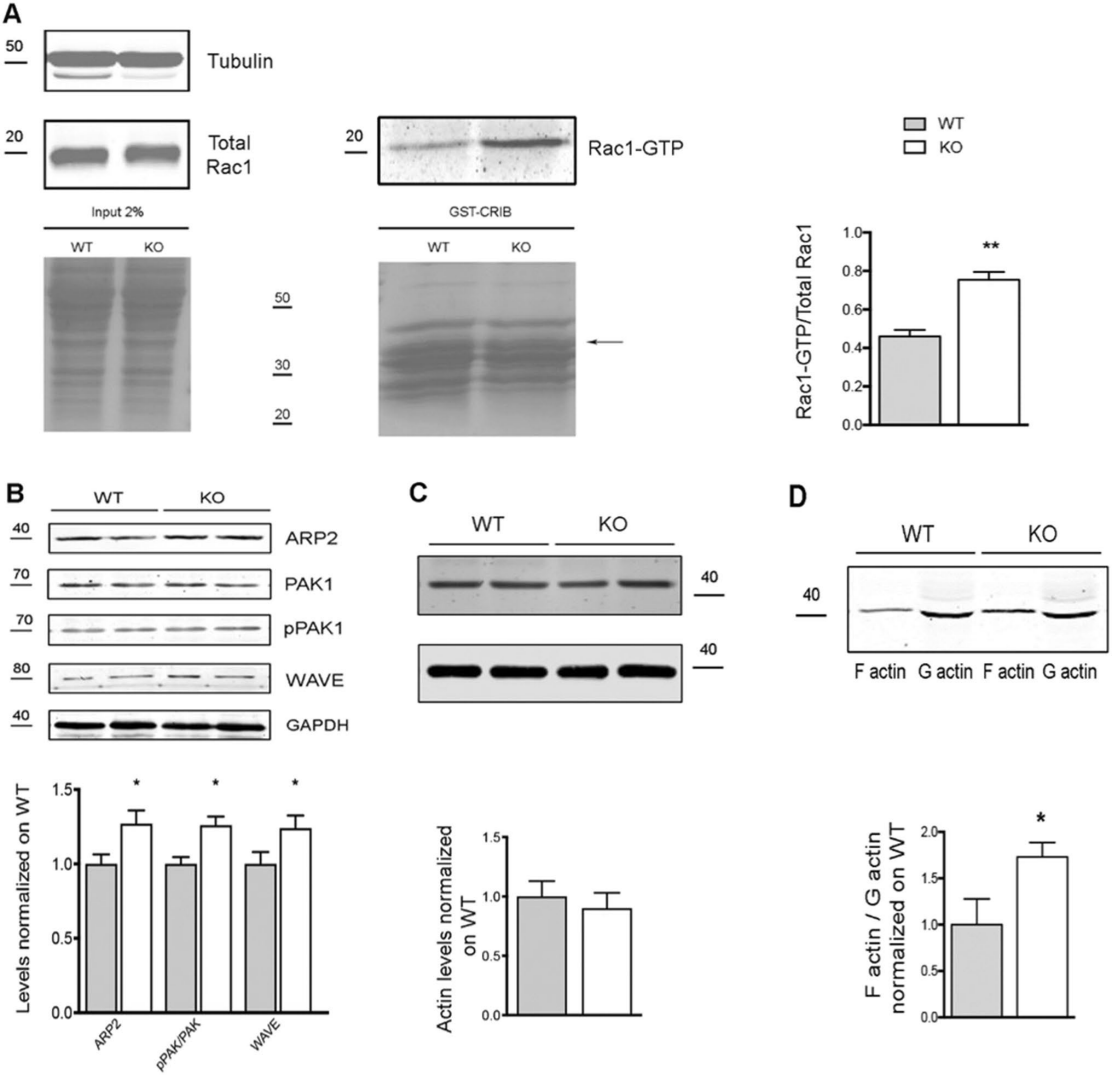
*Arhgap22* KO mice present hyper-active RAC1 pathway in the brain. **A** Activated RAC1 GST-pulldown on hippocampal and cortical lysates. Red ponceau show the amount of protein in the lysates and GST-CRIB beads used for the experiment. Western blot of total RAC1 (total Rac1) and active RAC1 (Rac1-GTP) are shown. Tubulin was used as internal control (**A**, top). (**A**, right histogram) Quantification of the level of active Rac1 normalized on total Rac1 protein. KO animals present elevated level of Rac1-GTP. **B** Western blot analyses on hippocampal and cortical lysates of WT and KO mice. Levels of RAC1 downstream effectors ARP2/3, WAVE, PAK1, and pPAK1 have been quantified and normalized on GAPDH (left panel). ARP2/3 and WAVE protein levels and the ratio between the active (phosphorylated) and total PAK1 are increased in KO mice compared to WT (right panel). Error bars indicate ± s.e.m. **C** Analyses of actin levels in cortical and hippocampal lysates from Arhgap22 KO and WT mice. Representative Western blot image (left) and quantification (right) indicate no changes between genotypes. Error bars indicate ± s.e.m. **D** Analyses of filamentous (F) and globular actin (G) ratio in cortical and hippocampal lysates from Arhgap22 KO and WT mice. Representative Western blot image (left) and quantification (right) indicate that KO animals presented higher F-actin/G-actin ratio compared to WT. Error bars indicate ± s.e.m

Considering that ARHGAP22 was shown to selectively inhibit RAC1 in other cellular systems [19], we wondered if the protein had the same function in the brain*.* Therefore, we performed a GST-pulldown experiment to evaluate the levels of activated RAC1 (RAC1-GTP) in *arhgap22*-deficient mice compared to WT. Briefly, we incubated cortical and hippocampal lysates with the GST-tagged CRIB domain of PAK1 protein, known to selectively bind active RAC1-GTP [43, 44]. Western blot analysis showed that *arhgap22* KO mice presented higher levels of RAC1-GTP compared to WT mice (RAC1GTP/Total RAC1; WT 0.460 ± 0.033 vs KO 0.754 ± 0.041 *n*_mice_ = 3, Student *t* test ** *p* < 0.01) (Fig. 2A).

The strong increase in RAC1 activity prompted us to study the downstream RAC1 pathway analyzing the levels of different proteins positively regulated by RAC1 activity (ARP2, PAK1, and WAVE) in cortical and hippocampal synaptosomes from WT and KO animals. These data demonstrated that KO mice presented an increase in the total levels of WAVE (WT 1.000 ± 0.061 vs KO 1.24 ± 0.065 *n*_mice_ = 5, Student *t* test * *p* < 0.05) and ARP2 (WT 1.000 ± 0.054 vs KO 1.271 ± 0.089 n = 4_mice_, Student *t* test * *p* < 0.05) proteins and in the levels of active PAK1 (phosphorylated form) (ratio pPAK/PAK; WT 1.000 ± 0.072 vs KO 1.321 ± 0.085 *n*_mice_ = 5, Student *t* test * *p* < 0.05) (Fig. 2B).

Lastly, since the aforementioned proteins are involved in the polymerization of actin in neurons [45, 46], we first evaluated the total level of actin in cortical and hippocampal samples from WT and KO mice and found no statistical difference (Fig. 2C ); and then, we analyzed the ratio between the polymeric (F) and the monomeric (G) form of actin (F/G ratio) in cortex and hippocampus of WT and KO mice. This revealed a strong increase in the F/G ratio (F/G ratio; WT 1.000 ± 0.160 vs KO 1.730 ± 0.090; *n*_mice_ = 3, Student *t* test * *p* < 0.05) (Fig. 2D) in KO animals, suggesting an increased RAC1-mediated actin polymerization.

These results suggest that *arhgap22* KO mice present hyper-activated RAC1 which, in turn, might induce actin cytoskeleton defects.

### Arhgap22 KO Mice Present Increased Spine Density in Hippocampus

**Fig. 3:**
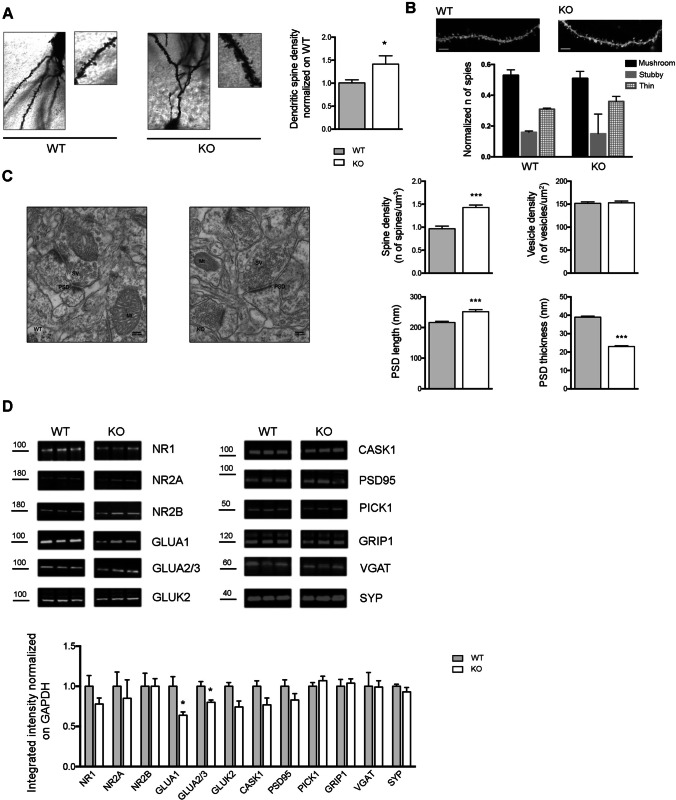
*Arhgap22* KO mice present increased spine density and altered synaptic molecular composition in hippocampus. **A** Representative Golgi staining images of hippocampal pyramidal neurons and high magnification of dendrites segments from WT (left) and KO (right) mice. Dendritic spine density measurement indicates increased number of dendritic spines in KO mice compared to WT mice. **B** Representative images of dendrites segments from WT (left) and KO (right) hippocampal neurons after DiI staining. Scale bar, 5 μm. No differences in spine morphology between genotypes are detectable. **C** Electron micrographs of asymmetrical synapses of the hippocampal CA1 regions of WT (left) and KO (right) mice. Scale bar, 100 nm. Analyses confirm increased spine density in KO mice and reveal alterations in PSD length and thickness. No pre-synaptic ultrastructural defects are detectable. The total surface area analyzed with stereology was 350 µm^2^ for each animal. **D** Representative Western blot images (top) and histograms (bottom) showing the quantification of synaptic markers on crude hippocampal and cortical synaptosomes from adult *arhgap22* WT and KO mice. Densitometry analyses performed with Li-Cor technology show that GLUA1 and GLUA2/3 subunits are significantly reduced in *arhgap22* KO mice compared to WT

The key role of RAC1 and actin cytoskeleton in the formation and maturation of dendritic spine is well established [47, 48]. Therefore, we evaluated the number of dendritic spines in hippocampal excitatory neurons in fixed brain slices of *arhgap22* KO mice by Golgi staining technique. Knockout animals presented a huge increase in the number of dendritic spines (WT 1.000 ± 0.065 vs KO 1.402 ± 0.18; *n*_neurons_ = 15–16, Student *t* test * *p* < 0.05,) (Fig. 3A). However, using this technique, it was not possible to evaluate the morphology of dendritic spines due to the limited resolution of light microscopy [49]. Consequently, in order to perform a morphological analysis of dendritic spines, we used the lipophilic fluorescent molecule DiI (1,1′-dioctadecyl-3,3,3′,3′-tetramethylindocarbocyanine perchlorate (“DiI”; DiIC18(3))) which, for its lateral diffusion properties, is able to stain neurons in fixed brain [50, 51]. We, then, performed DiI staining on brain slices of *arhgap22* WT and KO mice and analyzed hippocampal excitatory neurons at a confocal microscope. However, after confocal microscopy analysis, we did not find any alteration in spine morphology (Fig. 3B).

Afterwards, we studied the ultrastructure of hippocampal excitatory synapses through electron microscopy. Firstly, thanks to stereological analyses [28], we confirmed the increased dendritic spine density in KO mice (WT 0.967 ± 0.057 vs KO 1.430 ± 0.053, Student *t* test ** *p* < 0.01, with *n* = 3) (Fig. 3C). Moreover, we found that *arhgap22* KO post-synaptic density (PSD) was longer and thinner than WT animals (PSD length (nm), WT 216.25 ± 4.17 vs KO 251.55 ± 6.79; *n*_PSD_ = 180 *n*_mice_ = 3, Student *t* test *** *p* < 0.001; PSD thickness (nm), WT 38.97 ± 0.54 vs KO 23.03 ± 0.41; *n*_PSD_ = 180 *n*_mice_ = 3, Student *t* test *** *p* < 0.001) (Fig. 3C). On the pre-synaptic side, we did not find differences in vesicle density between the two genotypes (Fig. 3C).

In conclusion, these data suggest that the hyperactivation of RAC1 and the consequent actin cytoskeleton alterations might be responsible for PSD ultrastructural defects and increased dendritic spines density in *arhgap22* KO mice.

### Arhgap22-Deficient Synapses Present Altered Molecular Composition

Given the increased dendritic spine density and altered PSD, we asked whether ARHGAP22 absence could alter the molecular composition of synapses. To explore this, we analyzed crude synaptosomes from WT and KO mice by Western blot. We evaluated several synaptic markers including the following: glutamate receptors, PSD scaffold,proteins and pre-synaptic markers. We found reduced levels of GLUA1 (integrated intensity, WT 1.00 ± 0.121 vs KO 0.640 ± 0.041; *n*_mice_ = 4, Student *t* test * *p* < 0.05) and GLUA2/3 (integrated intensity, WT 1.000 ± 0.061 vs KO 0.800 ± 0.043; *n*_mice_ = 4, Student *t* test * *p* < 0.05) AMPA receptors subunits in *arhgap22* KO mice compared to WT littermates. No statistical differences for all the other proteins analyzed were detected, even if a tendency toward the decrease for NMDA receptor subunit NR1, GluK2 subunit of kainate receptor, CASK1, and PSD95 was present (Fig. 3D). All together, these data demonstrate altered dendritic spine molecular composition, suggesting an immature profile of *arhgap22* KO mice synapses.

### Arhgap22 KO Mice Display Reduced Neuronal Excitability

**Fig. 4:**
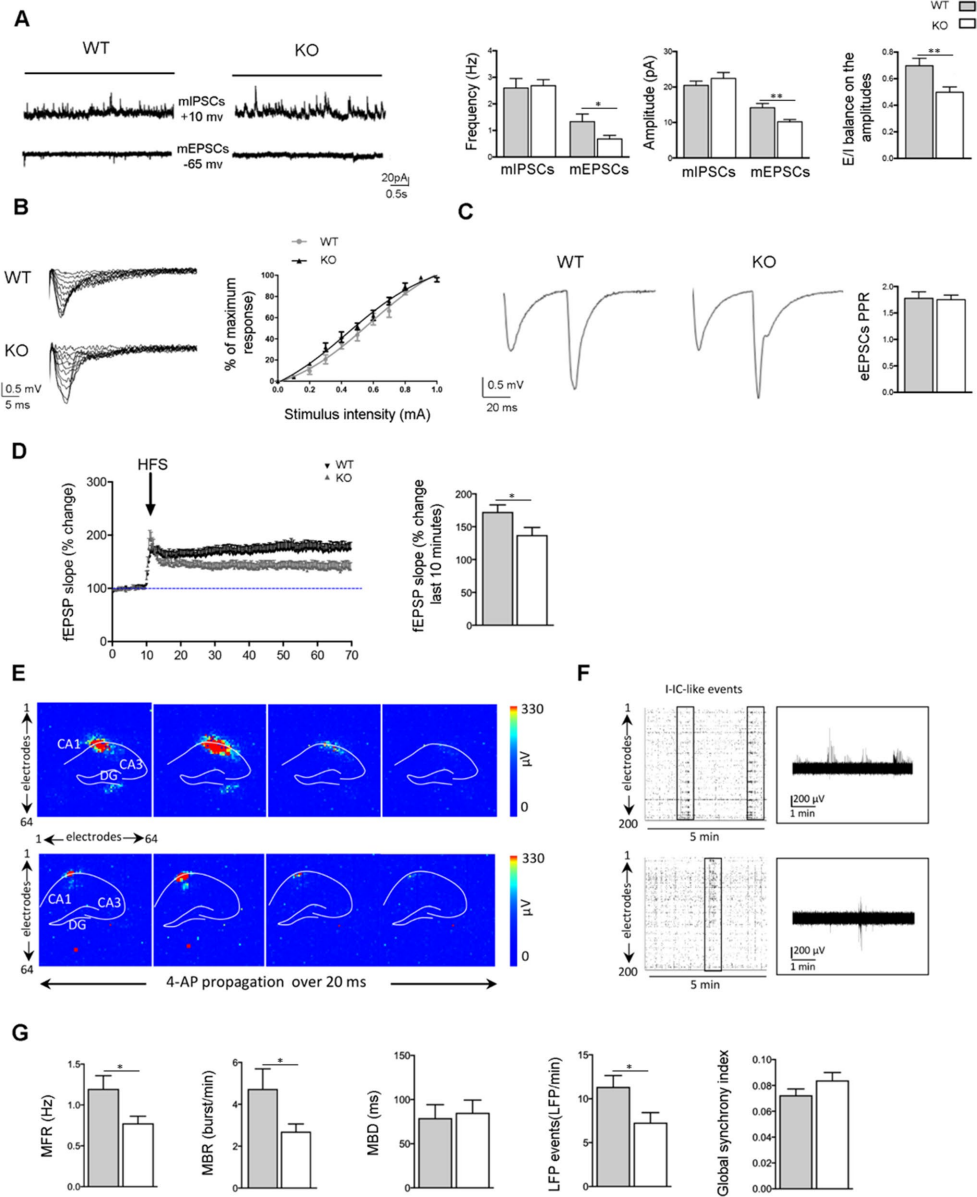
*Arhgap22* KO animals present altered excitation/inhibition balance, impaired LTP, and network activity. **A** Representative traces and quantification of mIPSCs (up) and mEPSCs (down) recorded from CA1 hippocampal pyramidal neurons from *arhgap22* WT and KO mice. Quantification (right panel) shows reduced frequency and amplitude of mEPSCs in KO animals while no alterations have been found for mIPSCs. These data reflect the impaired E/I balance in *arhgap22* KO mouse. **B** Representative traces of fEPSPs recorded from hippocampal CA1 of *arhgap22* WT and KO mice and quantification of the input/output relationship. **C** Representative traces and quantification of paired pulse ratio experiments showing no differences in the glutamate release probability between genotypes. **D** fEPSPs slope quantification before and after HFS shows impairment in LTP at Schaffer’s collaterals-CA1 synapses in *arhgap22* KO mice compared to WT. **E** Time laps representations of propagating events from representative WT (*top*) and Arhgap22 KO mice (*bottom*) slices upon 4AP (100 µM) chemical manipulation. Signals from 64 × 64-electrode array are represented in a false color map where each pixel shows the maximal signal variation of each microelectrode (µV). **F** 5 min raster plots of network hippocampal activity recorded by 200 channels from WT and KO mice. Each dot represents a detected spike, and each line is an electrode. Interictal-like activity is highlighted in black rectangles. **G** Quantification of mean firing rate (MFR), mean bursting rate (MBR), mean burst duration (MBD), LFP’s events, and Global Synchrony index for Arhgap22 WT, and KO mice are shown

Considering the modifications in spine density and in GLUA1 and GLUA2/3 AMPAR subunits expression, we investigated whether ARHGAP22 absence could induce alterations in Schaffer collateral (SC)-CA1 synaptic functions. Therefore, we recorded mEPSCs and mIPSCs from CA1 hippocampal pyramidal neurons in acute brain slices from *arhgap22* WT and KO animals. Interestingly, we found a significant decrease in mEPSC frequency and amplitude in KO mice (frequency (Hz), WT 1.333 ± 0.287 vs KO 0.678 ± 0.287; n_neurons_ = 17–18 *n*_mice_ = 3, Student *t* test *p < 0.05; amplitude (pA), WT 14.16 ± 1.21 vs KO 10.21 ± 0.63; n_neurons_ = 17–18 *n*_mice_ = 3, Student *t* test *p < 0.05) (Fig. 4A). On the other hand, we did not observe modifications in mIPSCs currents (Fig. 4A), suggesting that ARHGAP22 absence affects specifically the excitatory synapse.

A balanced excitatory and inhibitory (E/I) transmission is fundamental in the CNS, and our data suggested a disruption of this equilibrium in KO mice. As expected, *arhgap22* KO mice showed a decrease in the E/I ratio, calculated dividing the mEPSCs and mIPSCs amplitudes for every recorded neuron (E/I ratio, WT 0.6971 ± 0.0561 vs KO 0.4977 ± 0.0402 *n*_neurons_ = 16 *n*_mice_ = 3, Student *t* test ** *p* < 0.01) (Fig. 4A), suggesting a neurotransmission unbalance toward the inhibition.

Afterwards, we studied if the aforementioned alterations could affect synaptic plasticity in KO mice. To do this, we recorded field excitatory post-synaptic potentials (fEPSPs) at the SC-CA1 synapse in WT and KO mice, stimulating the SC and recording fEPSP from the *stratum radiatum* of the CA1 hippocampal region. Firstly, we generated an input/output (I/O) curve stimulating the SC with increasing intensity (0–1 mA) and analyzing the fEPSP slope in the CA1 as percentage of the maximal response. We did not found alteration for the I/O curve between genotypes (Fig. 4B). Since we found a decrease in mEPSCs frequency that could suggest an altered glutamate release probability, we performed paired-pulse ratio (PPR) experiments recording two fEPSP responses separated by 50 ms in WT and KO mice. The results demonstrated no changes for glutamate release probability in KO mice, confirming that ARHGAP22 acts mainly at the post-synapse (Fig. 4C).

Next, we investigated if *arhgap22* loss-of-function impaired also long-term potentiation (LTP) at SC-CA1 synapse. LTP was induced using a typical NMDA-dependent protocol (100 stimuli at 100 Hz) and fEPSP responses were recorded before (10 min baseline at half-maximal response) and after SC high-frequency stimulation. The analysis of fEPSP slope showed a significant LTP impairment in *arhgap22* KO mice compared to WT animals (last 10 min LTP (%), WT 172.6 ± 10.7 vs KO 137.4 ± 11.5 *n*_slices_ = 7–9 *n*_mice_ = 3, Student *t* test *p < 0.05) (Fig. 4D).

All together, these results suggested that ARHGAP22 absence causes significant defects in hippocampal excitatory synaptic function and plasticity, sparing the inhibitory transmission.

Finally, we investigated the hippocampal network activity from adult *arhgap22* KO and WT mice using a high-resolution microelectrode array (MEA) [30].

Since we did not observe any spontaneous activity under control conditions (aCSF, not shown), we induced spontaneous epileptiform discharges superfusing slices with aCSF containing 4-aminopyridine (4AP, 100 μM), a classical K^+^ channel blocker [52]. 4-AP is reported to enhance both inhibitory and excitatory synaptic transmission [53], representing a useful tool to uncover changes in excitability. Thus, *arhgap22* WT and KO mice hippocampal slices global activity was recorded onto MEA chips for 5 min (Fig. 4E). Visual inspection of the raster plots indicated that 4-AP elicited isolated spikes, single-channel bursts, and synchronous interictal (I-IC)-like discharges in both genotypes (Fig. 4F) [54]. In particular, I-IC-like discharges are considered to represent local field potentials (LFPs), and involve large neuronal populations in the hippocampus [55].

Specifically, we observed a significant decrease of mean firing rate (MFR (Hz), WT 1.19 ± 0.16 vs KO 0.79 ± 0.08; *n*_slices_ = 7–9 *n*_mice_ = 3; Student *t* test *p < 0.05) and mean bursting rate (MBR (burst/min), WT 4.73 ± 0.95 vs KO 2.69 ± 0.36 burst/min; *n*_slices_ = 7–9 *n*_mice_ = 3, Student *t* test *p < 0.05) in *arhgap22* KO mice compared to WT, while mean burst duration (MBD) was unaltered (Fig. 4G, first three left histograms). We also found that LFP events were decreased in KO mice compared to WT mice (LFP/min, WT 11.35 ± 1.29 vs KO 7.27 ± 1.14; *n*_slices_ = 7–9 *n*_mice_ = 3, Student *t* test *p < 0.05) (Fig. 4G, fourth histogram from the left). Furthermore, no difference in global synchronization of the hippocampal networks was observed between KO and WT mice (Fig. 4G, last histogram from the left). These results suggest that the hippocampal network of arhgap22 KO mice is less excitable than WT mice.

### Arhgap22 KO Mice Present Learning/Memory Defects and Reduced Anxiety-Like Behavior

Since we found different alterations at morphological, biochemical, and electrophysiological level, we hypothesized that *arhgap22* KO animals could present behavioral defects. In particular, considering the impairment in LTP, one of the fundamental mechanisms at the basis of learning and memory [56], we subjected *arhgap22* WT and KO mice to cognitive tests.

**Fig. 5:**
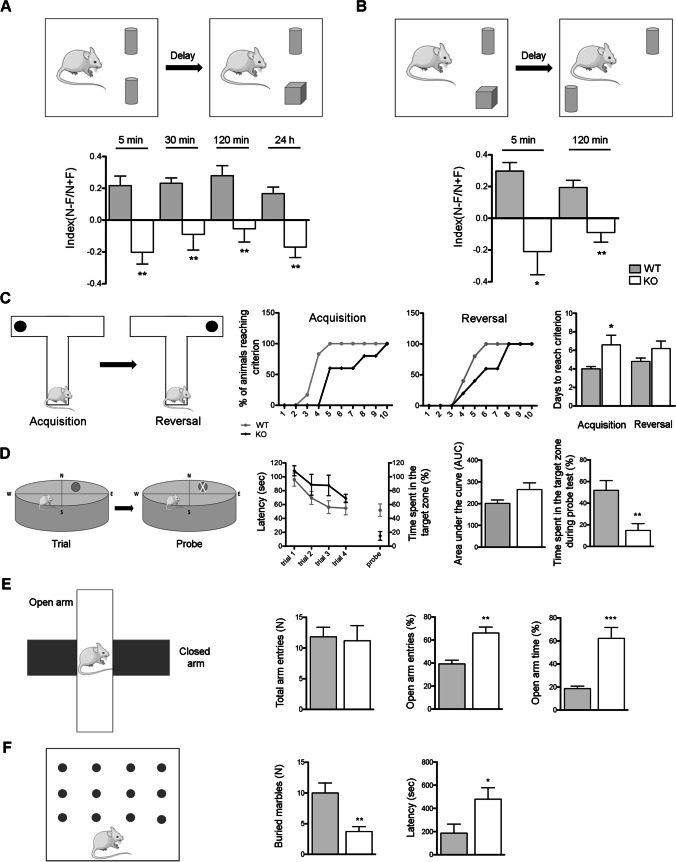
*Arhgap22* KO mice present learning/memory defects and reduced anxiety-like behaviors. **A**, **B** Representative scheme of the NOR test (**A**, top) and SOR test (**B**, top) are shown. *Arhgap22* KO mice present altered capability for episodic (novel object) and spatial memory (spatial object recognition). **C** Schematic representation of T-Maze test is shown. As indicated by quantification, *arhgap22* KO mice present a significant impairment during T-Maze acquisition phase but not in the reversal phase. **D** Scheme of Morris Water maze test during trial and probe phase. *Arhgap22* KO mice spent more time trying to reach the target zone during the trial phase. **E** Representative scheme of elevated plus maze test (left). Time spent in the open arm and number of entries in the open arm indicate that KO mice are less anxious than WT mice as shown by quantification. **F** Representative scheme of marble-burying test (left). Marble-burying test presents reduced number of marbles buried and an increase latency to the first burial

First, we tested *arhgap22* WT and KO littermates in novel object (NOR) and spatial object recognition tests (SOR). In NOR, KO mice presented a significant reduction of the discrimination index between the old and the new object compared to WT at all the time-points analyzed (index 5 min (N-F/N + F), WT 0.2167 ± 0.0606 vs KO − 0.2025 ± 0.0741; index 30 min (N-F/N + F), WT 0.2320 ± 0.0330 vs KO − 0.0900 ± 0.0987; index 120 min (N-F/N + F), WT 0.2790 ± 0.0638 vs KO, − 0.0540 ± 0.0840; *n*_mice_ = 10 per group, two-way ANOVA **p < 0.01) (Fig. 5A). In particular, the discrimination index can vary between + 1 and − 1. Positive scores indicate that the animals spent more time with the novel object while negative scores indicate preference for the familiar ones and values approaching zero indicate a null preference [57]. The preference for the novel object demonstrate that the familiar object is present in mice memory [58]; on the contrary, a null preference indicates that mice interact by chance with the two objects, suggesting that they forgot the previously encountered object, and thus reflecting a memory impairment. Accordingly, our analysis demonstrated that KO mice discrimination index values, even if negative, are not statistical different respect to zero, suggesting that KO mice interact randomly with the two objects. On the contrary, WT mice showed positive values statistically significant at all the time points tested, indicating a preference for the new object. In SOR, KO animals failed to discriminate between two objects based on spatial cues both at 5 and 120 min after familiarization (index 5 min (N-F/N + F), WT 0.2975 ± 0.0536 vs KO − 0.2100 ± 0.1460; index 120 min (N-F/N + F), WT 0.1938 ± 0.0454 vs KO − 0.0900 ± 0.06071; *n*_mice_ = 9 per group, two-way ANOVA *p < 0.05, **p < 0.01) (Fig. 5B). These results indicate that alteration in hippocampal functions in *arhgap22* KO mice are reflected by defects in recognition and spatial learning and/or memory.

Spatial learning and memory were additionally analyzed through T-maze test. Also, in this case, we found differences between the two genotypes; indeed, *arhgap22* KO mice showed an increased latency to reach the criterion in the acquisition phase compared to WT (days, WT 4.00 ± 0.03 vs KO 6.60 ± 1.03; *n*_mice_ = 6 per group, two-way ANOVA with Bonferroni post hoc test *p < 0.05) (Fig. 5C). In contrast, no differences were found during the reversal phase although a trend was detectable.

We therefore subjected mice to the water maze test, a hippocampus-dependent paradigm [59]. Knock-out mice showed an upward trend in the latency to find the platform during the trial phase. During the probe test, *arhgap22* KO mice spent less time in the target quadrant compared to WT mice (time in the target (%), WT 51.875 ± 0.048331 vs KO 14.82 ± 6.2982; *n*_mice_ = 6 per group, Student *t* test **p < 0.01) (Fig. 5D).

Since the hippocampus has been reported to influence the innate anxiety behavior [60], we analyzed also this aspect in our mice. We therefore subjected the mice to two commonly used tests: elevated plus maze (EPM) and marble-burying tests. In EPM, KO mice entered more times (open arm entries (N), WT 39.330 ± 3.199 vs KO 66.180 ± 5.117; *n*_mice_ = 6 per group, Student *t* test **p < 0.01) and spent more time in the open arm compared to WT (open arm time (%), 18.600 ± 2.185 vs KO, 62.270 ± 9.474; *n*_mice_ = 6 per group, Student *t* test ***p < 0.001) (Fig. 5E). No differences in the total number of entries were detected. Lastly, in marble-burying test, KO animals buried fewer marble stones (buried marbles (N), WT 10.000 ± 1.633 vs KO 3.714 ± 0.8081 *n*_mice_ = 7 per group, Student *t* test **p < 0.01) and presented an increased latency to bury compared to WT littermates (latency (s), WT 186.500 ± 77.690 vs KO 479.700 ± 98.520 *n*_mice_ = 6 per group, Student *t* test *p < 0.05) (Fig. 5F).

These data demonstrate that *arhgap22* KO mice present impairment in learning and memory formation and reduced anxiety-like behavior.

### Treatment with Rac1 Inhibitor NSC23766 Rescued Synaptic Alterations in arhgap22 KO Mice

**Fig. 6:**
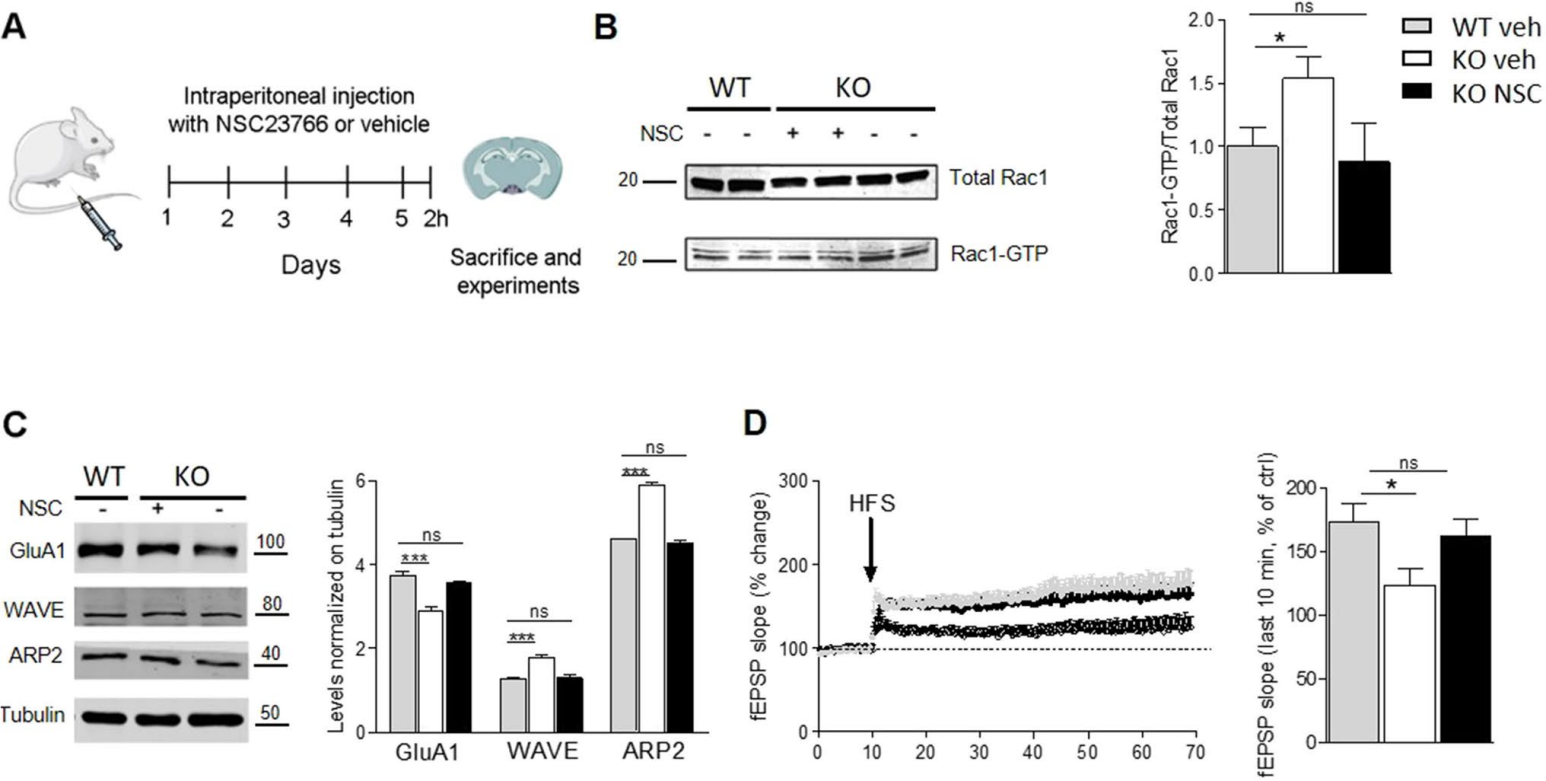
RAC1 inhibition by NSC23766 ameliorates the synaptic phenotype in *Arhgap22* KO mice. **A** Representative scheme of pharmacologic treatment. **B** Western blot (left) and quantification (right) showing the normalization of RAC1 activity after NSC treatment in KO mice. **C** Western blot (left) and quantification (right) showing the normalization of GluA1, WAVE, and ARP2 protein expression after NSC treatment in KO mice. **D** Quantification of LTP induction upon RAC1 activity normalization showing the full recovery of synaptic plasticity in KO mice

Next, we investigated whether treatment with an inhibitor of RAC1 could rescue the defects observed in *arhgap22* KO mice For this purpose, we evaluated synaptic functions after treatment with NSC23766, a compound previously reported to inhibit RAC1 activity [61]. The chronic treatment with NSC23766 (2 mg/kg of NSC23766 or vehicle once a day for 5 days) induced a normalization of RAC1 activity in KO mice (Fig. 6A, B). Noteworthy, vehicle-treated KO mice showed increased level of Rac1-GTP, supporting the specificity of our result. The NSC23766 treatment completely restored the normal protein expression level of GluA1 subunit of AMPA receptor as well as WAVE and ARP2, but not of GluA2/3 AMPA receptor subunit (Fig. 6C and data not shown). Moreover, the drug administration induced full recovery of LTP in KO mice (Fig. 6D).

All together, these results demonstrated that RAC1 activity normalization was able to restore some of the defects showed by KO mice.

## Discussion

This work stems from two main previous observations: (1) ARHGAP22 regulates RAC1 activity in cancer cell models; and (2) RAC1 is a key player in controlling actin cytoskeleton dynamics in neurons. These evidences prompted us to investigate whether ARHGAP22-mediated regulation of RAC1 in neurons could coordinate synapse formation and plasticity, given the importance of actin cytoskeleton dynamics in these processes.

We observed that ARHGAP22 is present in several mouse tissues [18] including the brain, where is predominantly expressed in cortex and hippocampus. This is not surprising, since ARHGAP22 protein was previously detected in the post-synaptic compartment of cultured rat hippocampal neurons [21]. Interestingly, ARHGAP22 reaches the maximum expression level at P14, when synaptogenesis peaks [42], similarly to some RAC1 GEF and GAP proteins [62, 63]. Moreover, in *arhgap22* KO mice, we found a hyper-activity of RAC1 and an upregulation of ARP2, PAK1, and WAVE, proteins known to be involved in neuronal actin dynamics [45, 46]. Coherently, in absence of ARHGAP22, we found an increased F/G actin ratio and, as expected, an increased hippocampal dendritic spine density.

Generally, an increased number of dendritic spines positively correlates with an increase in AMPAR content. Surprisingly, in KO mice, we found a decrease in AMPAR subunits expression. On the other hand, several studies have demonstrated that actin dynamic is critical for AMPAR trafficking and function [64]. Therefore, we can speculate that the reduction in Arhgap22 KO mice AMPAR subunits expression could be the effect of an impaired exocytosis, endocytosis, and/or endosomal recycling that makes dendritic spines less functionally active.

Structurally, we found that KO mice present a roughly 40% reduction in the PSD thickness. This parameter has been suggested to depend on protein translocation from cytoplasm to PSD [65] and on AMPA receptors content, with smaller PSD containing less AMPA receptors and vice versa [66–69]. This result might support the hypothesis of impairment in AMPAR trafficking. It is widely accepted that neuronal post-synaptic function depends on post-synaptic receptor density and/or efficacy. Coherently, in *arhgap22* KO mice, we found decreased glutamatergic currents, while the inhibitory transmission was unchanged, suggesting that ARHGAP22 absence affects specifically the excitatory synapse. In accordance, we found an unbalance toward inhibition in *arhgap22* KO mice. Interestingly, *arhgap22* KO mice showed decreased LTP at the SC-CA1 synapse. A similar effect was reported in mice after the hippocampal expression of a constitutively active form of RAC1 [70]. This result might be explained by two main hypotheses: (1) the reduction in AMPAR subunits impairs the removal of the Mg^2+^ block on NMDA receptor, which activation is crucial for LTP induction [26]; and/or (2) a defect in the dendritic spines structural plasticity [71], driven by the increased RAC1-mediated actin polymerization. Since a proper actin dynamic is essential for spinogenesis [48, 72], synaptic plasticity [71], and AMPAR trafficking [64], it is likely that in *arghap22* KO mice, dendritic spines, even if increased in number, are in a structural and molecular “crystallized” conformation due to the strong increase in actin polymerization. This phenomenon would make dendritic spines less dynamic and less responsive to stimuli, both during formation and plasticity-induced reorganization.

As discussed above, patch-clamp data suggested an altered E/I balance in *arhgap22* KO mice. However, despite being the gold standard for studying neuronal function, patch-clamp gives information about a limited number of neurons and offers poor spatial resolution. To integrate patch-clamp results in a more circuit-based view, we performed MEA experiments using high electrode density probes (4096 electrodes). We found a decreased activity in terms of mean firing and bursting rate in *arhgap22* KO mice hippocampus, suggesting a hypo-functionality of the circuit and supporting the neuronal E/I unbalance. Additionally, we also found that *arhgap22* KO mice present a decrease in the frequency of LFP insurgence. Interestingly, blocking ionotropic glutamatergic receptors decreases amplitude and frequency of LFPs [73]; we can therefore speculate that the reduced LFP frequency in our model might be the result of the diminished AMPA-dependent glutamatergic activity. Furthermore, being the LFP, a summation of excitatory and inhibitory signals of a large neuronal population, it is also reasonable to assume that an impaired E/I balance could induce alteration in LFP.

Alterations in hippocampal synaptic structure and function are often reported in animal models of intellectual disability that, in turn, present impairments in learning and memory behaviors [26, 74, 75]. Concordantly, when *arhgap22* KO and WT mice were subjected to a battery of behavioral test, we found that the former presented strong impairments in learning and memory formation. This is not surprising, since several proteins with RHOGAP activity, such as OLIGOPHRENIN1, ARHGAP15, and ARHGAP33 for instance, have been linked to higher cognitive functions [76–78]. In addition, an upregulation of RAC1 activity has also been found in fragile X syndrome, which is characterized by cognitive dysfunctions, aberrant plasticity, and immature dendritic spines [79], partially mimicking what we found in *arhgap22* KO.

Moreover, we also observed a significant decrease in anxiety-like behaviors in *arhgap22* KO mice. This is in accordance with evidence demonstrating that a disequilibrium between excitation and inhibition might be responsible for anxiety [80]. In particular, it has been demonstrated that GABA_A_ receptors antagonism increases anxiety-like behaviors while blocking AMPA receptors induces an opposite effect [80], resembling what we found in *arhgap22* KO mice.

We hypothesized RAC1 hyper-activity as leading cause of the KO mice defects, then we tried to pharmacologically inhibit RAC1 activity in our model. Interestingly, treating KO mice with NSC23766 drug, previously reported to inhibit RAC1 activity (56), we were able to rescue some of the KO mice defects. In particular, we found that NSC23766 treatment of KO mice re-established RAC1 activity, and restored the expression of GluA1 AMPAR subunit as well as WAVE and ARP2. Importantly, RAC1 inhibition fully recovered LTP.

In conclusion, our results shed light on ARHGAP22 protein as an important player in excitatory synapse formation and function as well as in cognition. These activities are likely to be orchestrated through the regulation of RAC1-mediated actin dynamics since we demonstrated that normalizing RAC1 activity rescued several defects in KO mice, supporting RAC1 activity modulation as a promising approach for cognitive dysfunctions.

## Data Availability

The authors confirm that the data supporting the findings of this study are available within the article and are available on request from the corresponding authors, LM and MP.
